# Texturing Methods of Abrasive Grinding Wheels: A Systematic Review

**DOI:** 10.3390/ma15228044

**Published:** 2022-11-14

**Authors:** Sharlane Costa, Mário Pereira, João Ribeiro, Delfim Soares

**Affiliations:** 1 Center for Microelectromechanical Systems (CMEMS), University of Minho, 4800-058 Guimarães, Portugal; 2 LABBELS—Associate Laboratory, 4710-057 Braga, Portugal; 3 Centro de Investigação de Montanha (CIMO), Instituto Politécnico de Bragança, Campus de Santa Apolónia, 5300-253 Bragança, Portugal; 4 CF-UM-UP, Centro de Física, Universidades do Minho e Porto, 4710-057 Braga, Portugal; 5 Instituto Politécnico de Bragança, Campus de Santa Apolónia, 5300-253 Bragança, Portugal; 6 Laboratório Associado para a Sustentabilidade e Tecnologia em Regiões de Montanha (SusTEC), Instituto Politécnico de Bragança, Campus de Santa Apolónia, 5300-253 Bragança, Portugal

**Keywords:** textured grinding wheel, grooved grinding wheel, texturing method, grooves, grinding wheel

## Abstract

Creating textures on abrasive wheels is a strategy that allows a significant improvement in grinding operations. The reduction of the internal stresses in the workpiece and the temperature during the grinding operation generates an increase in the dimensional accuracy of the workpiece and a longer tool life. Textured abrasive wheels can be produced in many different ways. Depending on the processing method, the dimensional accuracy of the tool and its applicability is changed. Some methods can produce tools with three-dimensional grooves; there are also methods that are employed for the re-texturing of grooves after the grooved zone wears out. In the literature, the benefits of textured grinding wheels over traditional wheels have been extensively discussed. However, information on the particularities of texturing methods is still lacking. To clarify the advantages, limitations, and main advances regarding each of the groove production methods, the authors of this article carried out a systematic review. The objective of this work is to establish the factors that are affected by groove production methods and the technological advances in this area. The benefits and drawbacks of various grooving techniques are then reviewed, and potential study areas are indicated.

## 1. Introduction

Abrasive grinding wheels are widely used in industry for the roughing and surface finishing of components through a chip-removal process [1,2]. This requires very high cutting forces which, together with the friction between the tool and the workpiece to be worn, generate high amounts of heat [3,4]. Excess heat can cause several problems, both in the part to be machined—a poor surface finish and change to its microstructure, in particular—and in the tool, especially its premature wear [5,6].

Conventional grinding wheels are basically composed of abrasive particles and bonding materials. The bonding agent binds the particles in place and gives the wheel its form and structure [7]. In addition to abrasive grains and bonding substance, grinding wheels also contain air spaces or pores. These pores help the transport of the refrigerant fluid and, therefore, in the removal of heat; however, as they are distributed randomly, only 40% of the applied fluids reach the work surface [8]. The topography of the grinding wheel affects the cutting performance and, consequently, the economic efficiency of a grinding operation [9,10]. The texture shaping on the surface of a grinding wheel in order to improve its performance and effectively minimize the use of coolants has been a very active field of research.

Textured abrasive grinding wheels are among the most promising abrasive tools, particularly for difficult-to-cut materials, such as ceramic matrix composites [11], hard–brittle materials [12], and carbon-fiber-reinforced polymers [13]. Compared to other new techniques, such as soft abrasive polishing [14,15], grooved wheels have a wide range of applications in the removal of materials, with improved grinding performances compared to conventional abrasive tools and the ability to generate structured surfaces in a controlled manner [16]. Utilizing texturized grinding wheels with straight and spiral type grooves reduced surface roughness by 4–5 times for Cu, 1.5 times for brass, and 3 times for Al6061 compared to traditional wheels [17,18].

Several patents referring to “slotted”, “textured”, or “grooved” wheels [19,20,21] indicate that interest in grooved grinding wheels began in the 20th century [19,20,21]. In the 1970s, helical grooves were formed on the surface of a grinding wheel using a crushing roller dressing tool [22]. This sparked academic interest in grooved wheels. There are now several ways of grooving grinding wheels. Lasers or CNC (computer numerical control)-actuated single-point diamond dressing tools and selective laser sintering have been developed as more sophisticated methods in recent years. Nonetheless, more robust methods, such as crushing roll dresser/profiler and prefabricated grooving methods, are still in use.

Textured wheels are defined by Li et al. [8] as tools with active and passive grinding zones on their geometrically active surfaces. These “passive” grinding sections are portions of the grinding wheel that do not come into contact with the workpiece and serve as a tank to deliver additional cutting fluids to a work zone and provide bigger chip-disposal spaces. Textures with different patterns, shapes, and sizes are produced on the work surfaces of these tools. The creation of textures on the surface of the grinding wheel can improve chip removal, increase heat dissipation, reduce surface damage, and decrease grinding force [23].

Regarding grooved wheels, there is a range of terminology employed in the literature. The terms “intermittent grinding wheel”, “structured grinding wheel”, “segmented grinding wheel”, “textured grinding wheel”, and “patterned grinding wheel” have been used to describe abrasive grinding wheels with grooves, slots, pockets in the surface, or gaps between abrasive segments in the case of single-layer tools that cause the wheel to make intermittent contact with the workpiece during grinding [8].

The advantages that can be obtained with the texturing of abrasive wheels have been documented by other authors [8,24], such as reductions in the process forces and the working temperature in the grinding zone. However, most authors have not presented comparisons between grooves produced by different processing methods. It is important to understand the limitations and advantages of each method, since the dimensional accuracy of grooves depends on how they are produced. The objective of this work is to establish the factors that are affected by particular groove production methods and the technological advances in this area.

Through a systematic review, this document classifies grooved wheels according to their manufacturing processes, namely, machined grooves, engineered grooves, laser grooves, 3D-printed grooves, segmented grooves, and grooves produced by the abrasive waterjet method. The works on each of the methods are organized in tables, making it possible to identify what has been studied by researchers more objectively.

## 2. Methods

A systematic review was carried out to identify what has been studied regarding the methods of producing grooves in abrasive wheels since 2010 and the main advantages and limitations of each one.

To obtain this information, an electronic search was performed in the Science Direct, Scopus, and Web of Science databases until July 2022, following the recommendations given in the PRISMA Statement [25]. The search strategy included studies with textured abrasive grinding wheels that informed which method was used to produce the grooves and their geometries. A broad search strategy was used, including the following keywords: “Textured abrasive tool”, “Textured grinding wheel”, “Grooved grinding wheel”, “Segmented grinding wheel”, “Patterned grinding wheel”, “Structured grinding wheel”, “Engineered grinding wheel”, Texture AND “grinding wheel”, Groove AND “grinding wheel”, and Channel AND “grinding wheel”. In addition, the authors’ database was used to include relevant works that were not found by searching for keywords in the online databases.

After removing duplicates, titles and abstracts were read and decontextualized articles were removed. For relevant publications or those with inadequate information in the abstract, the whole article was accessed. To be included, the articles had to detail how the grooves were produced by the authors. All texturing techniques were considered. The exclusion criteria for articles were: (1) publications before 2010; (2) review articles, systematic review articles, meta-analyses, book chapters, conference papers, patents, or reports; (3) studies not in English; (4) studies where the focus of the work was not the texturing of tools; (5) the grooving method was not clearly informed; and (6) the texturized tool was not a grinding wheel.

## 3. Results

**Figure 1:**
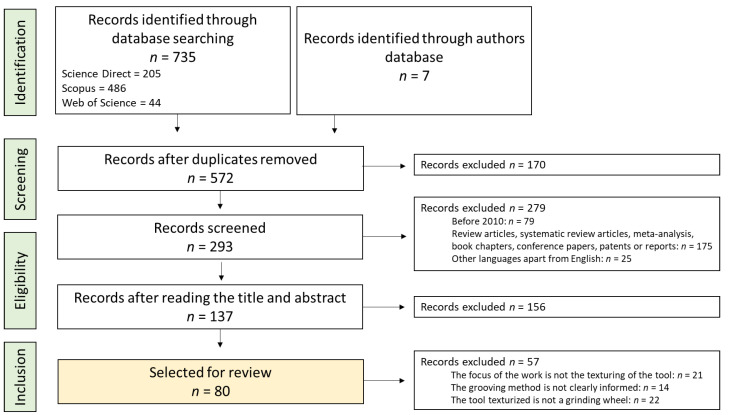
PRISMA flow diagram of the study selection process.

The electronic database search, last updated on 18 July 2022, yielded 205 hits from Science Direct, 486 hits from Scopus, and 44 hits from Web of Science. Seven articles from the authors’ database were included. One hundred and seventy duplicates were removed. After removing 79 works that were published before 2010, 175 that were not research articles (exclusion criterion 2), and 25 that were not in English, 293 studies were initially screened. The titles and abstracts of these works were read, and 156 were excluded due to decontextualization. Therefore, 137 studies were selected for full-text readings. These articles were further reviewed to determine eligibility, and 57 were excluded (according to criteria 4, 5, and 6), leaving 80 articles for evaluation in the systematic review. Figure 1 shows a flowchart of the selection procedure.

**Figure 2:**
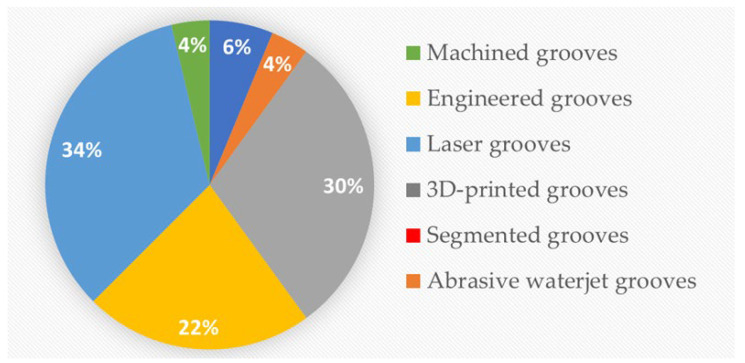
Distribution of works according to groove processing method.

**Figure 3:**
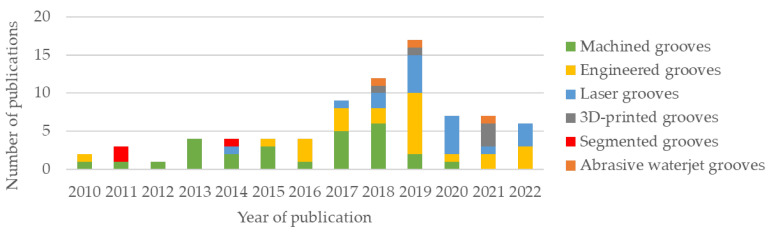
Distribution of works according to year of publication.

Of the papers included for review, 27 were on abrasive grinding wheels with machined grooves, 24 were on engineered grooves, 18 were on laser-beam-made grooves, 5 were on grooves produced using additive manufacturing techniques (3D-printed grooves), 3 featured the use of abrasive waterjet methods, and 3 were on segmented grooves. The distributions of works according to groove production method and according to year of publication are presented in Figure 2 and Figure 3, respectively.

From Figure 3, it can be seen that the number of publications on texturing methods for abrasive grinding wheels underwent a notable growth between 2016 and 2019. Machined grooves, despite having the highest number of publications in the analyzed period, since 2019 have given way to less conventional methods, such as laser and additive manufacturing, and also engineered grooves, with most publications dealing with numerical simulations and mathematical models.

## 4. Discussion

### 4.1. Machined Grooves

By machining a standard grinding wheel with another type of tool, such as a diamond disc, a milling cutter, or single- or multi-point tools, machined grooves can be formed. The machining tool is controlled manually, by an electromagnetic shaker or a servomotor. In other cases, the tool is static, and the grinding wheel movement is controlled.

**Figure 4:**
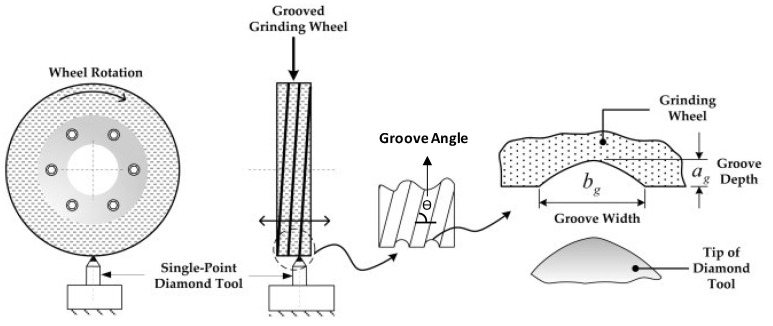
Production principle for machined grooves; groove patterns and geometries [26].

The principle of the method is shown in Figure 4, where, in this case, a single-point diamond dressing tool is used to create a helically formed groove on the working surface of the grinding wheel with a groove depth of $a_{g}$ and a groove width of $b_{g}$ [26]. The main disadvantage of the method is the wear of the grooving tool.

Table 1 summarizes the main subject of each published work on machined grooves; the groove geometries investigated in these works and the types of tools used for production are summarized in Table 2.

From Table 1, it can be gathered that the majority of the authors who study the machined groove method have published works evaluating the influence of groove parameters (such as angle, size, and geometry) on grinding operations. In addition, there are authors who have published novel grooving strategies using other types of tools to create grooves [50,52] or which involve the use of dedicated software, using the finite element method (FEM) to obtain the numerical vibration mode shape of the grinding wheel during texturing [46] or to develop test geometries in order to compensate for kinematic errors [48]. It is also clear that the use of mathematical models has gained attention in recent years. These models can be used, for example, to simulate the profiles of grooves both in the grinding wheel and in the profile generated in the workpiece [43,44].

**Figure 5:**
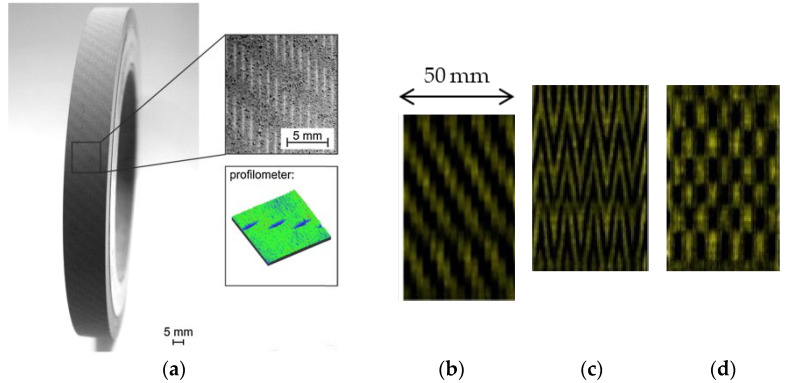
Machined grooves in (**a**) slit form [36], (**b**) helical form, (**c**) zigzag form, and (**d**) cross linear form [27].

In a general context, grooves produced by mechanical methods have simple geometries. Table 2 shows that the most commonly used geometry is the helical geometry, followed by slit geometries (discontinuous lines). Zigzag and linear cross-shaped grooves (like a mesh) were also produced. Although some authors have named the geometries of the grooves differently, in this article we sought to group geometrically similar grooves (not necessarily by the names given by the authors); for example, Silva et al. [42] described the shape of the grooves as “Round dimples and chevrons”; however, in the present paper, the grooves were classified as “slits and zigzag”, respectively. An example of each geometry is shown in Figure 5. The largest groove width produced by this method was 5 mm and the smallest was 0.0012 mm, while the depth varied between 5.0 and 0.002 mm.

**Figure 6:**
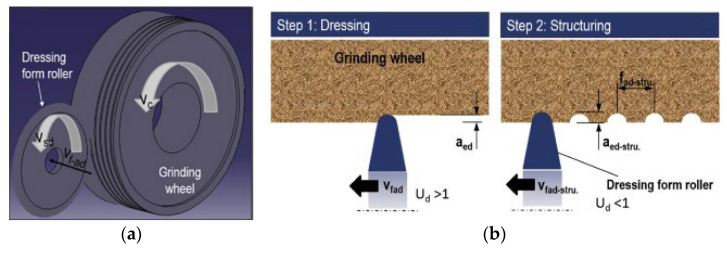
Illustration of the texturing process of the grinding wheel: (**a**) flattening the grinding wheel with standard dressing, followed by specific conditioning to form macrostructures on the wheel’s surface (**b**) [45].

Table 2 also shows that the most used tools for grooving are single-point tools and cutting discs. All grooves produced by cutting discs have helical geometries, except for the work carried out by Silva et al. [46], who produced grooves with a slit form on the surface of an abrasive wheel. Figure 6a illustrates the texturing process for a grinding wheel using a cutting disc (dressing form roller) [45]. The individual setup of the dressing kinematic parameters, including the form of the dresser, the dressing depth of cut $\left(a_{ed}\right)$, and the dressing feed $\left(f_{ad}\right)$, creates the required structures on the wheel surface. As shown in Figure 6b, the wheel is initially flattened under standard dressing conditions (step 1). The appropriate structure is then formed by treating the wheel surface with an overlap ratio less than one (Ud < 1) (step 2).

A cutting disc does not provide the accuracy and flexibility suitable for producing more complex grooves. Using a single-point diamond tool, it is possible to produce grooves with more detail, such as zigzag forms. Grooves produced with diamond tools, whether single-point or multi-point, have conical profiles characteristic of the tips of the grooving tools, as shown in Figure 4. However, to analyze groove width and depth separately, it is necessary to be able to generate a groove the width of which is independent of its depth. In order to create a rectangular section groove, Riebel et al. [52] adapted a polycrystalline diamond tool. These tools are manufactured with diamond grits inserted in a metallic matrix. The original had a cylindrical shape with a diameter of 3.2 mm, while the adapted tool was in the form of a “shank” or a rectangular prism, the 1.7 mm edge of which was used to groove a grinding wheel. The deepest grooves fashioned in the study took nearly 200 passes of the cutting tool to achieve their full depth; thus, any inaccuracy in position synchronization between the groover and the wheel would result in an error in the final groove geometry [52].

In general, machining is the most commonly used method among researchers, possibly due to the low costs and versatility. However, when the process is carried out manually, the finishing of the part is usually not well-controlled, so this process has been automated, which can make the method more expensive. The main disadvantage of the method is the intensive wear of the texturing tool. Most of the published work on machined grooves is aimed at evaluating the influence of groove parameters (such as angle, size, and geometry) on grinding operations. Despite being a simple and conventional method, new strategies, such as the application of software to compensate for kinematic errors, have also been shown to be a field of interest for the authors, as has the use of mathematical models.

### 4.2. Engineered Grooves

**Figure 7:**
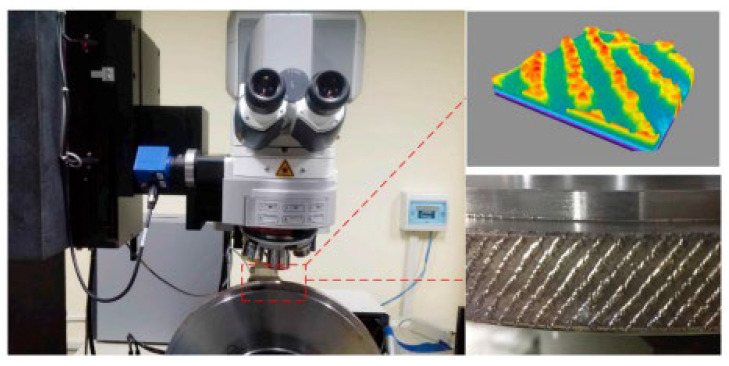
Topography of a monolayer abrasive grinding wheel with an arrangement pattern [53].

On monolayer grinding wheels, instead of removing grains on the wheel surface, it is possible to create grooves by placing the abrasive grits in a pre-defined pattern [8]. Grinding wheels with ordered grain distributions are called engineered wheels, where the texture depth is the height of the abrasive particle and the width is the space between each grain or agglomerate (Figure 7) [53]. Despite their inherent advantages in grinding, it is difficult to manufacture designed wheels with tiny grains because abrasive grains are always positioned manually or via the use of a template, which is strongly reliant on grain size [54].

The main subject of each published work on engineered grinding wheels is summarized in Table 3; the groove geometries investigated in these works are summarized in Table 4.

Table 3 shows that the main objective of the authors working with engineered grooves is the development of mathematical models and simulations capable of describing the grinding process. This type of approach models the grinding process to fully analyze the influence of various parameters and to describe, for example, the material removal mechanism, grain density, or grinding temperature [53,56,57]. By using these strategies, the number of experiments required can be significantly reduced. Furthermore, purely experimental methods only provide data at the end of grinding, for example, the surface topography of the finally generated part, but cannot reflect the material-removal mechanism in the machining process [55]. On the other hand, although in smaller numbers, there are authors carrying out purely experimental work, testing different geometries and comparing them with monolayer abrasive wheels with random grain distributions.

Engineered grooves are mostly studied on wheels with a defined abrasive arrangement in a single layer. Research on grinding wheels with 3D, controllable, abrasive arrangements is rarely mentioned. In the work developed by Qiu et al. [68], grinding wheels were designed with different 3D, controllable, abrasive arrangements in the space. Using a kinematic equation, the grinding trajectories were estimated in order to determine the effects of 3D abrasive configurations on the surface quality of the workpiece during the grinding process. To manufacture grinding wheels, a stereolithography equipment machine utilizing additive manufacturing technology was created. The influence of abrasive dispersion on the regularity of grinding trajectories was explored experimentally and computationally.

**Figure 8:**
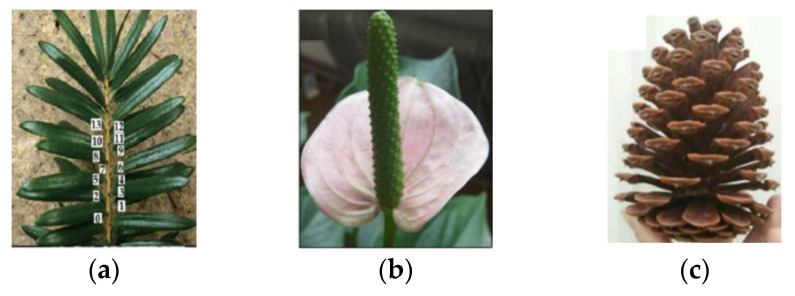
Typical examples of phyllotactic patterns: (**a**) keteleeria davidiana, (**b**) anthurium, and (**c**) pinecone [62].

From Table 4, it can be gleaned that the geometry most studied by the authors is the phyllotaxis type. Phyllotaxis is a kind of order that the leaves, fruit, and organizations of most plants conform to [77]. Figure 8 depicts typical examples of phyllotactic patterns [62]. Although the phyllotaxis idea has existed for more than a century, it has only recently been investigated by specialists and academics. The leaves or other natural organizations must have evolved patterns to withstand wind force to a certain degree, allowing plants to withstand wind. The phyllotactic pattern must be the pattern that allows wind and rain to move through the spaces between leaves more readily in order to lessen the influence of wind force. The characteristics of the phyllotactic pattern must meet the requirements of the designed grinding wheel with respect to the program (flow rate and speed, for example) and how the fluid is provided for the contact zone [62]. Therefore, authors who use this concept usually work with simulations and mathematical modeling, precisely to define the geometry chosen with accuracy and its potential gain.

**Figure 9:**
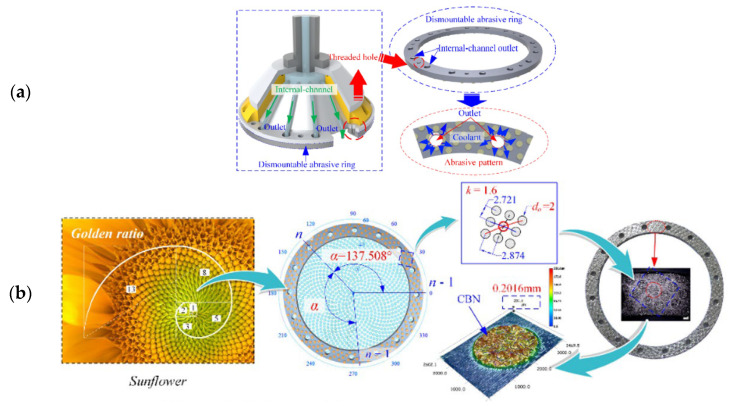
(**a**) Structure of the internal-cooling grooved grinding wheel and (**b**) the abrasive ring with the phyllotactic pattern of the abrasive [76].

Abrasive wheels with an internal cooling system have been studied by many authors in the last decade, the structure of which is shown in Figure 9a [76]. The coolant is injected directly into the grinding zone via the internal channel to prevent lubricant waste and energy consumption caused by the air barrier [72]. To further improve the coolant flow in the contact area with the workpiece, the authors have also produced engineered grooves in this type of abrasive grinding wheel. For example, in Figure 9b [76], the phyllotaxy concept was used to develop a sunflower texture on the grinding wheel’s abrasive ring.

Engineered grooves represent a field that has seen many publications since 2015. Researchers have paid special attention to the simulation and numerical modeling of abrasive particle arrays in order to continually improve the geometries that can be obtained using this method. Grinding wheels with 3D, controllable, abrasive arrangements may be a field of interest in the future, as the research carried out has resulted in only one publication reporting this type of engineered groove. The main disadvantage of the method is the time required to construct the arrangement, which is often achieved grain by grain.

### 4.3. Laser Grooves

**Figure 10:**
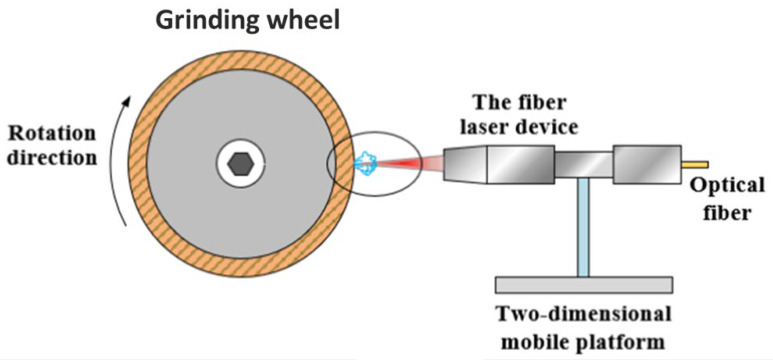
Experimental setup for laser structuring [81].

Laser conditioning is a novel, non-contact approach that may be used with a wide range of abrasives and bonding materials [78]. Laser-based thermal procedures utilize high-energy-density laser beams to ablate particular regions on abrasive tool surfaces so that materials within designed textural regions can be eliminated by melting, heating, vaporization, plasma generation, ablation of grits and bonds, or evaporation [79,80]. A common experimental setup for laser structuring is show in Figure 10 [81]. The primary advantages of the laser approach are the absence of tool wear, excellent reproducibility and controllability, high accuracy, and relatively quick processing time. In addition, this approach permits the microtexturing of grinding wheels [78].

The main subject of each publication on laser grooves is summarized in Table 5; the respective groove geometries and dimensions investigated are presented in Table 6.

**Figure 11:**
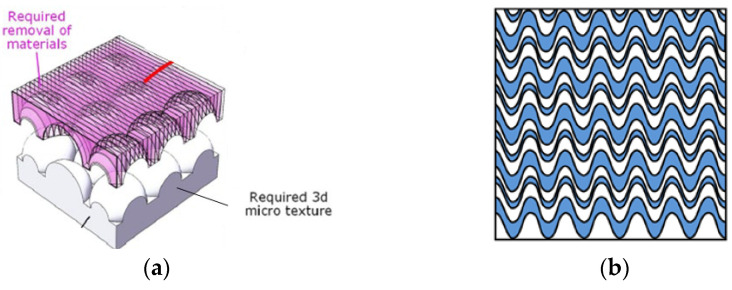
Groove geometries produced by the laser method: (**a**) hemispheres and (**b**) waves [80,86].

From Table 5, the evaluation of the influence of groove parameters on grinding operations is the area with the most publications. The data presented in Table 6 complement this information, as it is possible to find a wide range of different geometries. With the laser method, it is possible to produce macro- and microtextures from simple geometries, such as “helical” geometries, to more complex geometries, such as “waves” and “hemispheres”, as shown in Figure 11 [80,86].

**Figure 12:**
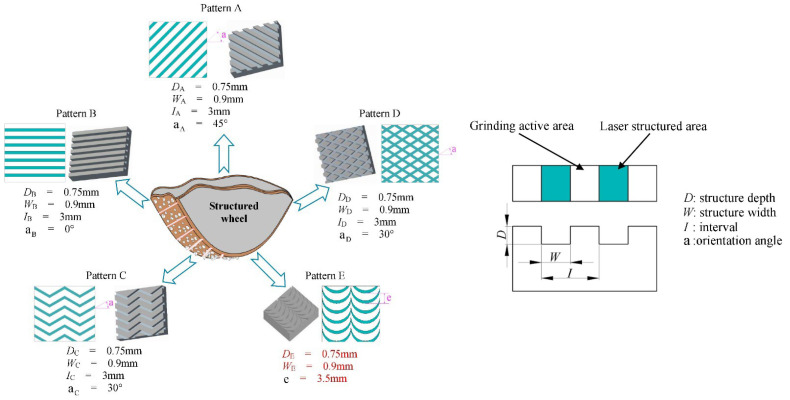
Different laser groove patterns discussed by Zhang et al. [87].

Some authors performed quite complete works from the point of view of studying groove parameters. Zhang et al. [84] produced six different structured grinding wheels. Different wheel speeds, depths of cut, and constant feed rates were utilized in a series of studies to determine their effects on the grinding performance of the wheel. The grinding forces were compared, and the impacts of wheel speed, depth of cut, and feed rate on grinding force were studied. In addition, the different characteristics of grinding wheel wear and surface roughness were compared. In another work developed by Zhang et al. [87], five different textures were produced (Figure 12). In addition to evaluating grinding forces and roughness, grinding temperatures were also discussed. Monier et al. [96] proposed a simulation approach for modeling textured wheels and their corresponding structured surfaces under a variety of operating situations. Regular and irregular geometries were studied, varying the spacing, angle, and dimension of the segments. However, grinding tests were performed only for one simple geometry (slot form).

In recent years, researchers have also offered methodological innovations. To separate the incubation impact of adjacent scanning, Hou et al. [97] developed an alternating laser scanning approach paired with staggered forward and backward traces with double pitch offset. The effects of ultrafast laser ablation on grinding wheel groove morphologies were investigated. In engineering practice, the laser machining model suggested by Geng et al. [98] offers a condition for the superabrasive grinding wheel with more efficiency and adaptability. A nanosecond pulse laser was incorporated into an ultra-precision machine tool and utilized for in-line grinding wheel conditioning, dressing, and texturing. To create precise profiles on the grinding wheel surface, an offset compensation approach taking into account the fluctuating depth of focus at different laser irradiation positions was developed [97,98].

**Figure 13:**
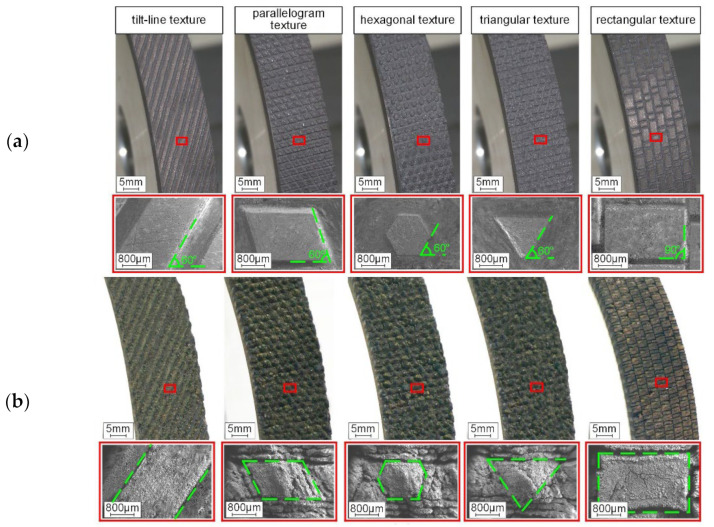
Texture appearance obtained using (**a**) appropriate and (**b**) inappropriate parameters [92].

Potential problems still need to be defined, such as the adjustment of texturing parameters, high energy consumption, and surface thermal damage [8]. To fill this gap, Li et al. [92] studied the effects of varying parameters of the continuous wave CO_2_ laser method on the production of five different textures on the surfaces of diamond abrasive tools. The author highlighted the importance of proper parameter selection. The results are shown in Figure 13. It is noteworthy that, unlike all other works, Li et al. [92] used a continuous wave laser, instead of a pulsed one.

The predominant benefits of this technology are no tool wear, excellent repeatability and control, high accuracy, and a very quick process time. In addition, this approach permits the microtexturing of grinding wheels. On the other hand, laser groove manufacturing requires the fine tuning of laser beam parameters before texturing and high power consumption control and entails high equipment maintenance costs.

### 4.4. 3D-Printed Grooves

The emergence of 3D printing technology enabled the fabrication of grinding wheels with intricate porous structures in a novel manner. Except for work developed by Qiu et al. [68], who produced a multi-layer engineered abrasive wheel, additive manufacturing is the only method that presents the possibility of texturing a wheel to the fullest extent, eliminating the need to refashion grooves after wear.

Grooved grinding wheels produced by this method can be obtained in different ways. Laser-assisted 3D printing systems employ high-powered laser beams to sinter or fuse consecutive cross sections of material to create a product, while DIW (direct ink writing) is an extrusion-based, heat-free approach, in which a ceramic ink can be extruded through a nozzle and form the desired structure [99]. Table 7 summarizes the work developed in this area; Table 8 specifies the groove geometries and the 3D printing methods used.

**Figure 14:**
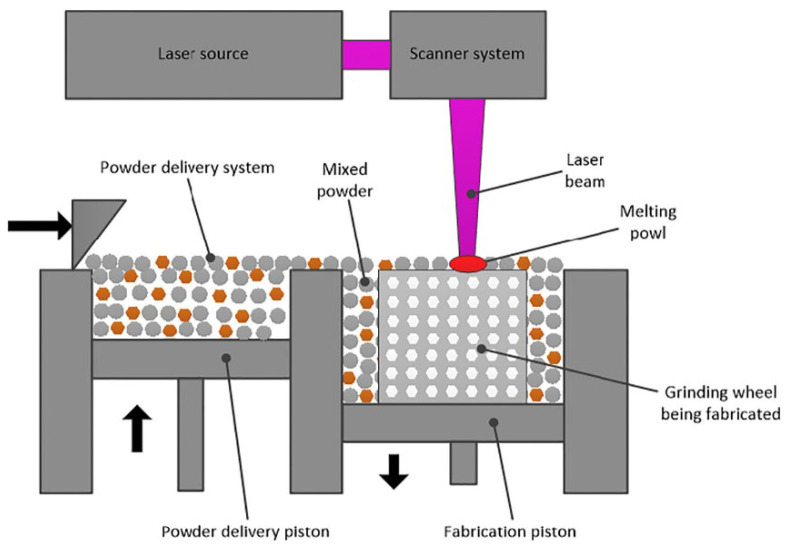
Principle of the SLM process [100].

**Figure 15:**
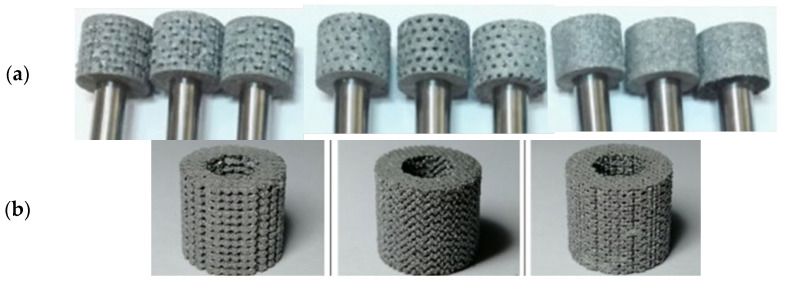
Abrasive wheels structured by the SLM method by (**a**) Tian et al. [100] and (**b**) Li et al. [103].

The SLM manufacturing principle adopted by some authors [100,101,102,103] is represented in Figure 14 [100], and some texturized grinding wheels that have been produced are shown in Figure 15 [100,103]. The SLM device has a powder supply system and a laser scanning system. The laser beam will selectively melt the AlSi10Mg powder layer by layer in accordance with the CAD data. The molten alloy powder will then solidify around the diamond abrasive grain to form the grinding wheel’s bond. The manufacturing is conducted in an inert atmosphere, and the wheel head is assembled on an aluminum substrate. Using wire electro-discharge machining, the wheel head is separated from the substrate following manufacture.

The disadvantage of the SLM method is that, due to the temperatures involved, graphitization of diamond grains may occur. Furthermore, as the laser melts the powders into a layer-by-layer mass, some inhomogeneity among the layers may result [99].

**Figure 16:**
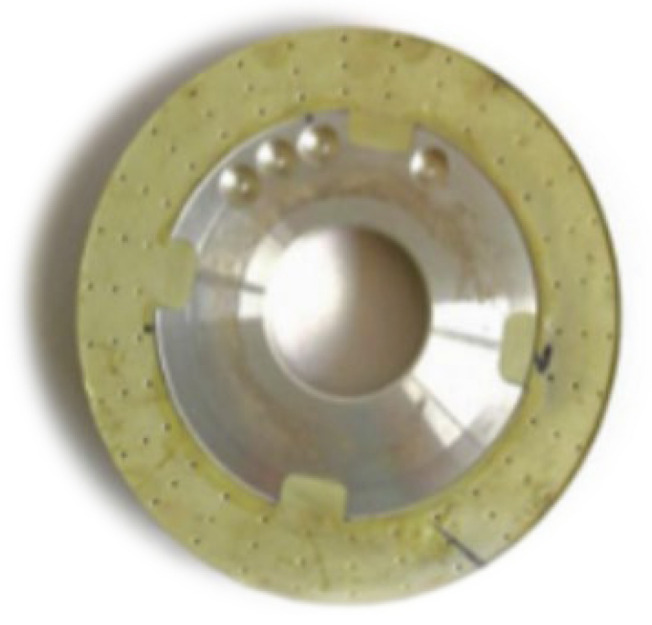
Abrasive grinding wheel with internal cooling holes produced by the SLS method [101].

The SLS technique developed by Du et al. [101] to produce structured abrasive wheels is likewise based on additive layer-by-layer manufacture. The nylon powder used for the 3D-printed grooves has not been scanned and sintered. After SLS, the untreated nylon powder is lost and flows out, leaving grooves like those shown in Figure 16. According to the authors, it is impossible to create internal linked holes with diameters of less than 1.5 mm. When printing smaller holes, the holes will get blocked, and internal channels cannot be generated [101].

**Figure 17:**
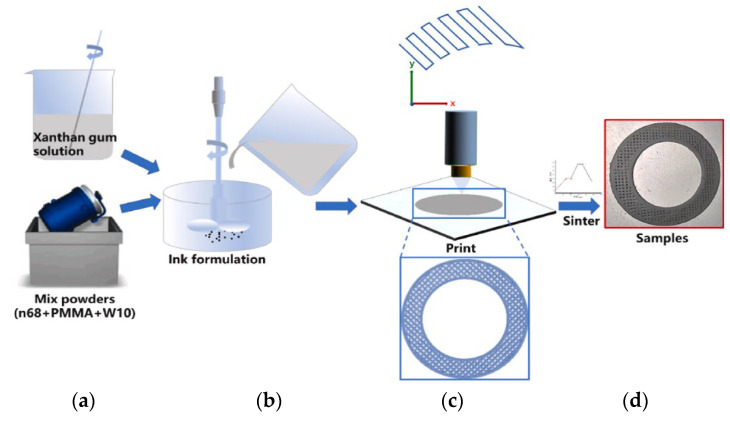
Fabrication procedure of the diamond grinding wheel by DIW: (**a**) mixing of components, (**b**) formulation, (**c**) printing and (**d**) final appearance of the sample [99].

The manufacturing method proposed by Huang et al. [99] and the appearance of the grinding wheels are presented in Figure 17. The DIW method consists, first, in preparing a ceramic ink by dissolving xanthan gum in water and then mixing it with n68 (vitrified bond powder), polymethyl methacrylate (PMMA—as the pore former), and diamond powder (W10), previously homogenized (Figure 17a,b). Then, as shown in Figure 17c, the ceramic ink is extruded by a direct ink writing machine. After the sintering process, the manufactured grinding wheels are cooled in an oven. A final sample can be seen in Figure 17d.

Additive manufacturing technology can be used in a variety of ways to build textured abrasive wheels. Despite being a method that requires a lot of time and resources, it can produce three-dimensional grooves, eliminating the need for tool reconditioning.

### 4.5. Segmented Grooves

**Figure 18:**
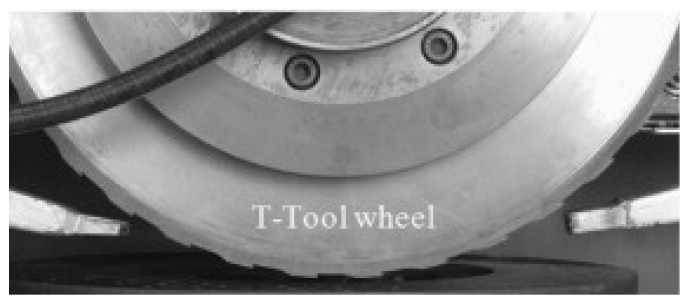
A segmented grinding wheel [104].

Grinding wheels with segmented grooves are produced by including segments in the grinding wheel’s manufacturing mold. The grooves will have the shape of the segments present in the mold. Once the grooves are completely worn out, it is not possible, by this method, to produce them again. An example of a segmented grinding wheel is shown in Figure 18 [104].

Table 9 summarizes the main subject of each published work on segmented wheels.

From Table 9, it can be seen that the works carried out using this method compare different aspects of the grinding operation of a segmented grinding wheel with the operation of a conventional wheel. All works used the same groove geometry, shown in Figure 18, with an intermittent ratio of 0.5. The authors did not provide detailed information on the abrasive wheel manufacturing method.

### 4.6. Abrasive Waterjet

**Figure 19:**
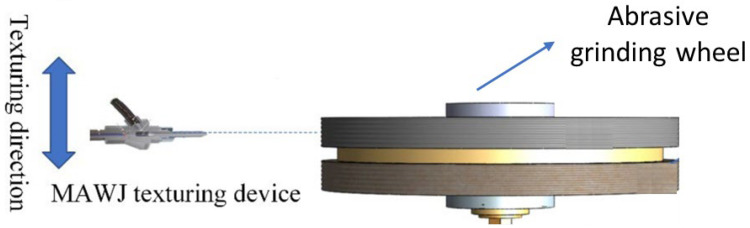
Illustration of the texturing procedure by microabrasive waterjet [108].

Abrasive waterjet (AWJ) machining is regarded as a cold material removal technique with great potential for the very effective dressing of grinding wheels [107]. As shown schematically in Figure 19, the high-velocity abrasive waterjet is targeted and injected in a radial mode at a standoff distance from the abrasive waterjet nozzle to the target grinding wheel [108]. Table 10 summarizes the works in this area; Table 11 specifies the studied groove geometries.

**Figure 20:**
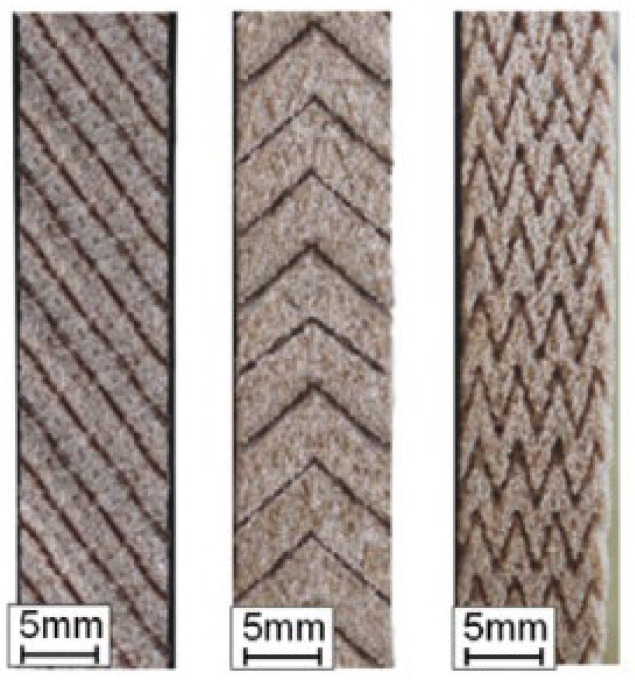
Grooves produced by the AWJ method [109].

The low dressing accuracy associated with AWJ micromachining technology is a significant obstacle to its employment in the production of grinding wheels with precise grooves [111]. Therefore, the authors who study this method, as can be seen in Table 10, seek to optimize the precision of the groove production process. From Table 11, it can be seen that the geometries used are relatively simple, although it is possible to produce discontinuous grooves with angular shapes, as shown in Figure 20 [109].

## 5. Conclusions

The conventional grinding process is a technique widely used to machine metals, ceramics, superalloys, and difficult-to-cut materials. However, the conventional geometries of grinding wheels do not favor the dissipation of the heat generated during grinding, and large amounts of the applied coolants are not used in the processes because they do not reach the work surfaces. Limited heat dissipation can cause excessive tool wear, increased grinding forces, residual stress, phase transformation, lower material removal rates, surface defects, and other kinds of thermal damage. To minimize these problems, textured abrasive tools were proposed. The production of grooves in abrasive tools can be achieved using several techniques. In this regard, machined grooves, engineered grooves, laser grooves, 3D-printed grooves, segmented grooves, and abrasive waterjet grooves were investigated in this paper.

The objective of this work was to establish the factors that are affected by the various groove production methods and the technological advances in the area. The main conclusions of this systematic literature review were:

(a) In recent years, new texturing methods have gained attention (such as additive manufacturing production techniques), while the number of publications on machined grooves has been decreasing since 2018.
(b) Among all the methods evaluated in this work, the engineered groove process is the one that has been demonstrated to be able to produce the most complex grooves. However, the application of the method is limited to monolayer grinding wheels. For wheels that are not monolayers, the laser method is the most suitable for producing grooves with greater geometric control. Furthermore, unlike machining techniques, the laser method has the advantage that the tool that produces the grooves does not wear out.
(c) In general, methods for making textured abrasive wheels are constantly in evolution; more and more innovative geometries and techniques have been studied and proposed. However, there is still a marked gap in the manufacture of abrasive wheels with three-dimensional channel structures, showing the technological difficulty. Almost all the techniques used until now only produce superficial grooves, that is, after the grinding wheel wears out, a new operation is necessary to refashion the grooves. Additive manufacturing is a method capable of producing internal channels; however, it is an expensive process and without industrial application feasibility for series production.
(d) For future work, it is suggested to explore the AWJ method, which, like the laser method, has the advantage of the absence of tool wear; however, there are still few publications on the subject. Three-dimensional grooves also represent a very promising field for future work. A technique capable of producing three-dimensional, textured, abrasive wheels in a simple and controlled manner has not yet been developed.

## Figures and Tables

**Table 1:** Summary of publications on machined grooves.

Author and Year	Summary
Oliveira et al., (2010) [27]	The work presented a texturing method based on an electro-mechanical exciter connected to the dressing tool which receives a synchronized signal from a control program that engraves patterns on the grinding wheel.
Gavas et al., (2011) [28]	A study of the influence of grooves and work material type on workpiece surface roughness was developed.
Tawakoli et al., (2011) [29]	Dry and wet grinding with grooved grinding wheels with 25, 50, and 100% active areas was evaluated.
Aurich et al., (2013) [30]	Evaluation of three types of grinding wheel: with internal lubricant supply channels and with and without channels and external lubrication.
Mohamed et al., (2013) [26]	A single-point diamond dressing instrument was used to cut grinding wheels with a shallow circumferential groove, with different active areas (50, 70, and 100%).
Nadolny et al., (2013) [31]	The kinematic parameters of microdiscontinuities were presented. The construction of a particular device that enables the obtainment of macrodiscontinuities with specific and defined surface shapes was also detailed.
Silva et al., (2013) [32]	A characterization and dimensional evaluation of the textures produced by the method described by Oliveira et al. [27] was presented.
Köklü et al., (2014) [33]	Study of the surface and dimensional qualities generated by varied-angle grooves as well as one of the key metrics of surface integrity residual stress.
Mohamed et al., (2014) [34]	A grinding wheel grooving method that can both groove and re-groove a grinding wheel was presented.
Gavas et al., (2015) [35]	The influence of different helically angled grinding wheels on surface roughness and roundness in diverse workpieces.
Denkena et al., (2015a) [36]	A novel approach for grinding wheel structures was presented. The approach is founded on the kinematics of fly cutting.
Denkena et al., (2015b) [37]	Presented a novel approach for the patterning of grinding wheels. The designs were manufactured with a patterning tool equipped with one to four diamonds and a normal dressing spindle.
Wójcik et al., (2017) [38]	A study of the influence of the grinding wheel modification (grooves) on workpiece surface roughness and residual stresses.
Azarhoushang et al., (2017) [39]	Grooved grinding wheel for dry grinding in three different conditions (30, 60, and 75% passive area).
Caydas et al., (2017) [40]	The purpose of the study was to determine the effects of the number of helically grooved wheels, workpiece rotation speed, and depth of cut on surface roughness in cylindrical grinding.
Liu et al., (2017) [41]	An analytical model representing the textures of grooved wheels based on ridge width, ridge length, texture angle, and ridge function parameters was constructed.
Silva et al., (2017) [42]	Discussion of the possibilities and limitations of structuring surfaces using two kinds of grooved grinding wheels. Workpieces were structured using a patterned grinding wheel, specially conditioned during the dressing operation.
Mohamed et al., (2017) [43]	Investigation of the ability of circumferentially grooved grinding wheels to create parallel ridges on a workpiece.
Cao et al., (2018) [44]	The effects of dressing and grinding settings on a collection of microstructural texturing models were discussed. The structural surface characteristics and the corresponding theoretical models were validated by experimental results.
Daneshi et al., (2018) [45]	The mathematical modeling of the process kinematics was used to simulate cylindrical plunge grinding by structured wheels (with 30, 60, and 70% passive area). Attention was given to the problem of transferring the texture from the grinding wheel to the workpiece.
Silva et al., (2018) [46]	Describes the creation of a patterning system for grinding wheels based on modal vibration analysis. The design, modeling, and simulation of the texturing device were described.
Dewar et al., (2018) [47]	The performances of non-grooved and grooved vitrified grinding wheels were compared using cylindrical plunge grinding trials. The grooving method used was the one developed in [34].
Forbrigger et al., (2018a) [48]	A profile grinding wheel grooving robot attachment was created, as well as a mechanism for assessing and adjusting for the robot’s kinematic error.
Forbrigger et al., (2018b) [49]	Helical grooves were used on profile grinding wheels. A generalized approach for computing the grooving factor for any grooved/textured profile grinding wheel was also presented.
Denkena et al., (2019) [50]	The wear behavior of chemical-vapor-deposited thick-film diamonds (CVD-Ds) in patterning grinding wheels was studied. CVD-D inserts were utilized as the structuring tool’s cutting edges.
Patel et al., (2019) [51]	Using cylindrical plunge grinding with grooved and non-grooved wheels, the relationship between workpiece surface roughness and speed ratio was carefully investigated.
Riebel et al., (2020) [52]	An experimental study of the relationship between grinding wheel groove depth and width in relation to grinding performance.

**Table 2:** Grooving tools and machined groove geometries and dimensions.

Author and Year	Grooving Tool	Groove Geometry			
		Form	Angle (deg)	Depth (mm)	Width (mm)
Oliveira et al., (2010) [27]	Single-point	Helical, zigzag, and cross linear	-	0.002–0.025	2
Gavas et al., (2011) [28]	Disc	Helical	90.52	3	5
Tawakoli et al., (2011) [29]	Disc	Helical	90	0.1	0.6
Aurich et al., (2013) [30]	Milling cutter	Slits	15	-	-
Mohamed et al., (2013) [26]	Single-point	Helical	-	0.1	0.5–1.08
Nadolny et al., (2013) [31]	Single-point	Helical	-	0.5	1.7
Silva et al., (2013) [32]	Single-point	Slits	-	0.005	1.5
Köklü et al., (2014) [33]	Disc	Helical	15, 30, 45	3	2.6
Mohamed et al., (2014) [34]	Multi-point	Helical	-	0.1–0.36	0.92–1.60
Gavas et al., (2015) [35]	Disc	Helical	15, 30, 46	3	2.6
Denkena et al., (2015a) [36]	Multi-point	Slits	-	0.020–0.2	0.2–0.5
Denkena et al., (2015b) [37]	Single-point and multi-point	Slits	-	0.020–0.1	0.2–0.5
Wójcik et al., (2017) [38]	Disc	Helical	15	4	3
Azarhoushang et al., (2017) [39]	Disc	Helical	-	~0.0058–0.0135	~0.2640–0.353
Caydas et al., (2017) [40]	Disc	Helical	45	3	3
Liu et al., (2017) [41]	Single-point	Helical	5.16	1.55	~0.1
Silva et al., (2017) [42]	Single-point	Slits and zigzag	-	0.005	0.587–10.35
Mohamed et al., (2017) [43]	Single-point	Helical	10–90	~0.005–0.025	~5
Cao et al., (2018) [44]	Multi-point	Helical	-	0.02–0.05	~0.12
Daneshi et al., (2018) [45]	Disc	Helical	-	~0.0135	~0.353
Silva et al., (2018) [46]	Disc	Slits	-	0.005	0.0012–0.006
Dewar et al., (2018) [47]	Single-point	Helical	90	0.102	0.884
Forbrigger et al., (2018a) [48]	Single-point	Helical	-	~5	5
Forbrigger et al., (2018b) [49]	Single-point	Helical	89.8	0.1–0.2	0.96–0.99
Denkena et al., (2019) [50]	CVD-D insert	Slits	-	-	0.156–0.488
Patel et al., (2019) [51]	Single-point	Helical	-	0.102	1.15

**Table 3:** Summary of publications on engineered grooves.

Author and Year	Summary
Yuan et al., (2010) [54]	An electroplated wheel with a controlled abrasive cluster was presented, and its performance in dry grinding carbono–epoxy composites was compared to that of a standard grinding wheel.
Wang et al., (2015) [55]	Models of the grinding wheel’s kinematics and elastic and plastic deformation were created. The surface topographies of three different engineered grinding wheels were also evaluated by numerical simulations.
Yu et al., (2016a) [56]	The dynamic cutting-point density model was constructed using a grinding wheel with an abrasive phyllotactic pattern, which may be applied to other manufactured grinding wheels with ordered, distributed abrasive grains.
Yu et al., (2016b) [57]	The surface roughness model using a grinding wheel with an abrasive phyllotactic pattern and a wheel with a general grain structure pattern was established. The model was also validated through experiments with various grinding parameters.
Yu et al., (2016c) [58]	The wear of abrasive particles on two differently designed grinding wheels was evaluated. One grinding wheel with a random distribution of grains was compared to an engineered one with a bio-inspired phyllotactic pattern.
Zhang et al., (2017) [59]	A spiral, orderly, distributed fiber tool was proposed. The flow field of cutting fluid was simulated by 3D fluid simulation software. Cutting experiments were used to validate the modeling results.
Ding et al., (2017) [60]	A model of surface topology reconstruction for the textured monolayer CBN wheels was established. Grinding-wheel wear experiments were conducted to investigate the evolution and influence of grain protrusion height nonuniformity in textured monolayer wheels.
Lyu et al., (2017) [61]	A mathematical model of the engineered grinding wheels was developed to predict the temperature in the grinding zone. The simulations were validated through surface grinding experiments with various grinding parameters.
Yu et al., (2018) [62]	The grinding fluid in the grinding zone was simulated for a bionic grinding wheel based on computational fluid dynamics (CFD) software.
Zhang et al., (2018) [53]	A wheel topography model was developed that can be integrated with a workpiece model, a kinematic model, and a calculation model of undeformed chip thickness of a single grain to obtain the distribution of undeformed chip thicknesses.
Yu et al., (2019a) [63]	Research and trials to determine the best grain arrangement on the grinding wheel to lower the grinding temperature were presented.
Yu et al., (2019b) [64]	A bionic structured surface inspired by phyllotaxis theory was created to reduce specific grinding energy in the grinding process.
Yu et al., (2019c) [65]	A bionic structured surface inspired by phyllotaxis theory was created to reduce grinding forces in the grinding process.
Zhang et al., (2019a) [66]	An integrated model based on the surface topography of an engineered grinding wheel was established. The grinding process was simulated, and the results were analyzed to obtain a surface roughness model and a specific grinding energy model based on the undeformed chip thickness distribution.
Zhang et al., (2019b) [67]	The effects of material removal rate, texture dimension, and radial dressing of grinding wheels on the distribution characteristics of undeformed chip thickness were studied through numerical and experimental analysis.
Qiu et al., (2019) [68]	On the basis of additive manufacturing technology, stereolithography apparatus equipment was created to manufacture resin-bonded grinding wheels with 3D programmable abrasive configurations.
Zhu et al., (2019) [69]	Wear and self-sharpening phenomena were evaluated and discussed for polycrystalline CBN grinding wheels with helically arranged grains.
Zhang et al., (2020) [70]	Patterned monolayer wheels were prepared. The effects of grit spacing, grit size, and arraying angle on grinding force, grinding temperature, and surface roughness were evaluated in a dry grinding operation.
Peng et al., (2020) [71]	An internal-coolant grooved wheel with various abrasive patterns was designed and prepared. The experiments explored the influence of abrasive patterns on grinding wheel performance.
Peng et al., (2021a) [72]	An internal-coolant grooved wheel was prepared. The CFD method was utilized to examine the flow field in the curved channel, together with the heat field and flow field in the grinding zone.
Peng et al., (2021b) [73]	An internal-coolant grooved wheel was prepared with a bionic phyllotaxis texture. The CFD approach was used to investigate the flow characteristics of the cup wheel with varied surface structures.
Guo et al., (2022) [74]	The grinding mechanism of engineered grinding tools (with different geometries) and a corresponding grinding force model were established.
Wang et al., (2022) [75]	A new simulation model was presented to predict the topography of the machined surface in superalloy grinding, considering the specific geometry and gesture of diamond grains.
Peng et al., (2022) [76]	A bowl-shaped grinding wheel with internal cooling and a phyllotactic abrasive pattern was designed. A CFD study optimized the grinding wheel’s interior structure.

**Table 4:** Engineered groove geometries and dimensions.

Author and Year	Groove Geometry		
	Form	Grain Size (mesh)	Width (mm)
Yuan et al., (2010) [54]	Phyllotactic	-	1.7
Wang et al., (2015) [55]	Rectangular	80	0.98
Yu et al., (2016a) [56]	Phyllotactic	-	~0.29–0.39
Yu et al., (2016b) [57]	Phyllotactic	70/80	~0.87–1.21
Yu et al., (2016c) [58]	Phyllotactic	70/80	~0.87–1.21
Zhang et al., (2017) [59]	Helical	-	1.6–2.9
Ding et al., (2017) [60]	Helical	80/100	1.2
Lyu et al., (2017) [61]	Phyllotactic	70/80	~0.87–1.21
Yu et al., (2018) [62]	Phyllotactic	-	~0.88–1.21
Zhang et al., (2018) [53]	Helical	40/50	2.5
Yu et al., (2019a) [63]	Phyllotactic	40/50	~0.75–1.06
Yu et al., (2019b) [64]	Phyllotactic	40/50	~0.75–1.06
Yu et al., (2019c) [65]	Phyllotactic	40/50	~0.75–1.06
Zhang et al., (2019a) [66]	Helical	40/50	1.2–2.4
Zhang et al., (2019b) [67]	Helical	40/50	1.2–2.4
Qiu et al., (2019) [68]	Spiral, rectangular, and circular	-	2.0–3
Zhu et al., (2019) [69]	Helical	80/100	1.2
Zhang et al., (2020) [70]	Rectangular	-	1.0–3
Peng et al., (2020) [71]	Helical and rectangular	80	1.5
Peng et al., (2021a) [72]	Helical	-	1
Peng et al., (2021b) [73]	Phyllotactic	80	-
Guo et al., (2022) [74]	Helical	-	~0.5
Wang et al., (2022) [75]	Helical	40/45	1.2
Peng et al., (2022) [76]	Phyllotactic	80	-

**Table 5:** Summary of publications on laser grooves.

Author and Year	Summary
Guo et al., (2014) [82]	The effects of laser parameters on microstructured surfaces (focal point shift, laser power, scanning speed, and scanning passes) were explored and adjusted.
Deng et al., (2017) [83]	The effects of groove angle and geometry on the surface quality of the workpiece were evaluated.
Zhang et al., (2018) [84]	Evaluation of six different groove geometries on grinding performance and wear behavior.
Guo et al., (2018) [85]	The effect of microgrooves combined with microrefinement of the abrasive grains on the surface of a CVD diamond wheel and on grinding force and grind quality.
Zhang et al., (2019a) [86]	To produce macro–microstructured patterns on the wheel’s surface, a methodical strategy was developed and implemented. A theoretical model of grinding force was provided and experimentally validated.
Zhang et al., (2019b) [87]	Evaluation of five different groove geometries on grinding operations. Grinding forces were compared and the influences of workpiece speed, grinding wheel speed, and depth of cut on grinding force were discussed.
Wu et al., (2019) [88]	Grooves were produced on an abrasive grinding wheel to later transfer the texture to the surface of hard and brittle working materials.
Azarhoushang et al., (2019) [89]	Evaluation of the grinding of ceramic matrix composites with grinding wheels structured by two different methods (laser and segmented). The grinding tests were carried out at different material-removal rates and cutting speeds. The laser method was more emphasized in the paper.
Deng et al., (2019) [90]	In a coarse-grained diamond grinding wheel, the groove shape, structuring efficiency, and rate of structured grains were all assessed.
Li et al., (2020a) [91]	Grinding wheels with inclined and rectangular-cross-section grooves were compared in the grinding process.
Li et al., (2020b) [92]	The influence of laser beam parameters on the generation of different groove geometries was studied.
Wu et al., (2020) [93]	Two different microtexture geometries were ablated directly on diamond grains on an engineered grinding wheel using a pulsed laser.
Zhang et al., (2020) [94]	A type of biomimetic fractal-branched grinding wheel was designed based on leaf veins. Grinding tests were performed and the experimental results were compared with those for a conventional grinding wheel.
Zhao et al., (2020) [95]	To produce patterned CBN/CuSnTi-grinding wheels, a coaxial powder feeding laser cladding technique utilizing CAD/CAM technology was introduced.
Li et al., (2021) [80]	The effects of laser pass numbers and scanning speeds on groove geometry were discussed.
Monier et al., (2022) [96]	The ability to pattern a wheel with advanced regular and irregular pattern geometries was evaluated by computational simulation. However, only a geometry was produced and tested in grinding.
Hou et al., (2022) [97]	A laser scanning method was used to create grooves on grinding wheels using an ultrafast laser processing device.
Geng et al., (2022) [98]	For metal-bond diamond grinding wheels, a conditioning approach was designed. The same laser source was used throughout the conditioning chain, including the truing, dressing, and texturing of the grinding wheel.

**Table 6:** Laser groove geometries and dimensions.

Author and Year	Groove Geometry			
	Form	Angle (deg)	Depth (mm)	Width (mm)
Guo et al., (2014) [82]	Helical	-	-	~0.004–0.016
Deng et al., (2017) [83]	“V” and “W” shapes	0, 30, 60 and 90	0.0382	0.0753
Zhang et al., (2018) [84]	Helical, cross linear, zigzag, and “U” shape	0, 30, 45 and 90	0.85	1.2
Guo et al., (2018) [85]	Helical	-	0.008	0.004–0.006
Zhang et al., (2019a) [86]	Cross linear, waves, and zig zag	30, 45 and 90	0.85	1.2
Zhang et al., (2019b) [87]	Helical, cross linear, zigzag, and waves	0, 30 and 45	0.75	0.9
Wu et al., (2019) [88]	Helical	4.52	~0.102	~0.187
Azarhoushang et al., (2019) [89]	Slots (segmented grooves) and cross linear (laser grooves)	-	0.35	0.85
Deng et al., (2019) [90]	“V” and “W” shapes	-	0.02–0.071	0.003–37.6
Li et al., (2020a) [91]	Helical	-	0.95 and 1	0.65
Li et al., (2020b) [92]	Helical, parallelogram, hexagonal, triangular, and rectangular	60 and 90	0.3–1	0.8–1.8
Wu et al., (2020) [93]	Cross linear, with and without holes	-	0.15–0.2	0.02–0.05
Zhang et al., (2020) [94]	Leaf vein	-	1	3
Zhao et al., (2020) [95]	Helical, zigzag, and “U” shape	-	-	-
Li et al., (2021) [80]	“V” shape and hemispheres	-	0.4–4	~0.18–0.3
Monier et al., (2022) [96]	Slots	-	-	~0.19–0.33
Hou et al., (2022) [97]	Lines with “U” shapes	-	1	1
Geng et al., (2022) [98]	Helical and cross linear	-	0.08–0.25	0.2–0.35

**Table 7:** Summary of publications on 3D printed grooves.

Author and Year	Summary
Tian et al., (2018) [100]	In this work, a novel approach for fabricating porous, metal-bonded grinding wheels using selective laser melting (SLM) technology was developed.
Du et al., (2019) [101]	In this investigation, resin-bonded diamond grinding wheels with internal cooling holes were 3D-printed using selective laser sintering (SLS).
Wang et al., (2021) [102]	The method described by Tian et al. [100] was used to produce four wheels with different groove geometries. The focus of the work was the grinding performance that could be achieved with different grinding wheels.
Li et al., (2021) [103]	The method described by Tian et al. [100] was used to produce three wheels with different groove geometries. Here, the focus was on the design and modeling of the different grinding wheels.
Huang et al., (2021) [99]	This work describes the use of direct ink writing (DIW) to manufacture three types of vitrified grinding wheels with varied groove geometries.

**Table 8:** Three-dimensional printing methods and geometries.

Author and Year	Method	Groove Geometry	
		Form	Width (mm)
Tian et al., (2018) [100]	SLM	Honeycomb and octahedron structure	-
Du et al., (2019) [101]	SLS	Holes	1.5 and 2.5
Wang et al., (2021) [102]	SLM	Octahedron, Schwarz P, Schwarz D, and Schoen I-WP structures	-
Li et al., (2021) [103]	SLM	Schwarz P, Schwarz D, and Schoen I-WP structures	-
Huang et al., (2021) [99]	DIW	Triangle and lattice	4 and 6

**Table 9:** Summary of the works on segmented grooves.

Author and Year	Summary
Tawakoli et al., (2011a) [105]	A segmented grinding wheel was designed, fabricated, and tested. The conventional grinding process was compared with the intermittent grinding process.
Tawakoli et al., (2011b) [104]	Study of the feasibility of intermittent grinding with a segmented wheel using two ceramic matrix composite (CMC) materials.
Azarhoushang et al., (2014) [106]	Study of the wear of non-segmented and segmented diamond wheels in high-speed deep grinding of carbon-fiber-reinforced ceramics.

**Table 10:** Summary of publications on grooves produced by AWJ.

Author and Year	Summary
Li et al., (2018) [109]	This research presented a method for designing groove shapes based on the intended operating temperature.
Zhang et al., (2019) [110]	This study aimed to estimate the essential profile characteristics, including groove depth and groove breadth, of the grooves on AWJ-textured metal-bonded grinding wheels.
Zhang et al., (2021) [111]	The mechanisms behind the AWJ truing process and the integrated rough–fine grinding process were studied to guide the development of the technology.

**Table 11:** Geometries and dimensions of the grooves produced by AWJ.

Author and Year	Groove Geometry			
	Form	Angle (deg)	Width (mm)	Depth (mm)
Li et al., (2018) [109]	Helical, zigzag, and “V” shape	31.4, 50.5, and 180	1.74 and 1.91	-
Zhang et al., (2019) [110]	Helical	90	~0.9–1.1	0.6–1
Zhang et al., (2021) [111]	Helical	90	1.6	0.5

## Data Availability

Not applicable.
